# A Compensatory Effect on Mate Selection? Importance of Auditory, Olfactory, and Tactile Cues in Partner Choice among Blind and Sighted Individuals

**DOI:** 10.1007/s10508-018-1156-0

**Published:** 2018-02-02

**Authors:** Agnieszka Sorokowska, Anna Oleszkiewicz, Piotr Sorokowski

**Affiliations:** 1 Smell and Taste Clinic, Department of Otorhinolaryngology, TU Dresden, Fetscherstr. 74, Haus 5, Keller, 01307 Dresden, Germany; 2 Institute of Psychology, University of Wroclaw, Wrocław, Poland

**Keywords:** Mate selection, Attractiveness, Blindness, Visual impairment, Sensory modalities

## Abstract

Human attractiveness is a potent social variable, and people assess their potential partners based on input from a range of sensory modalities. Among all sensory cues, visual signals are typically considered to be the most important and most salient source of information. However, it remains unclear how people without sight assess others. In the current study, we explored the relative importance of sensory modalities other than vision (smell, touch, and audition) in the assessment of same- and opposite-sex strangers. We specifically focused on possible sensory compensation in mate selection, defined as enhanced importance of modalities other than vision among blind individuals in their choice of potential partners. Data were obtained from a total of 119 participants, of whom 78 were blind people aged between 16 and 65 years (*M* = 42.4, SD = 12.6; 38 females) and a control sample of 41 sighted people aged between 20 and 64. As hypothesized, we observed a compensatory effect of blindness on auditory perception. Our data indicate that visual impairment increases the importance of audition in different types of social assessments for both sexes and in mate choice for blind men.

**Electronic supplementary material:**

The online version of this article (10.1007/s10508-018-1156-0) contains supplementary material, which is available to authorized users.

## Introduction

Human attractiveness is a potent social variable, and people typically assess their potential partners based on input from different sensory modalities, including visual (Cunningham, Barbee, & Pike, 1990; Fink & Penton-Voak, 2002), auditory (Collins & Missing, 2003; Feinberg, Jones, Little, Burt, & Perrett, 2005), and olfactory signals (Sorokowska, 2013; Wedekind, Seebeck, Bettens, & Paepke, 1995). However, among all sensory cues, visual signals are widely considered to be the most important and salient source of information about the social world, mainly because of their accessibility and their impact on the interpretation of information based on other inputs (Krupp, 2008). However, it is currently unclear how people who cannot see assess the attractiveness of others.

A large body of research has examined the sensory consequences of visual impairment, with various studies reporting that blind people possess superior tactile abilities, such as tactile discrimination (Goldreich & Kanics, 2003), and superior auditory skills, such as echolocation (Teng, Puri, & Whitney, 2012). In addition, brains of blind people have been found to respond more strongly to olfactory stimulation (Kupers et al., 2011). However, few studies have examined the effects of decreased visual input on social assessments. In the context of social olfactory perception, a small number of experiments have shown that both blind adults (Beaulieu-Lefebvre, Schneider, Kupers, & Ptito, 2011) and blind children (Ferdenzi, Coureaud, Camos, & Schaal, 2010) report stronger odor awareness than sighted peers, particularly in response to odors that naturally emanate from or are associated with human bodies. Further, congenitally blind people were reported to exhibit superior performance when discriminating negative emotions based on body odor samples (Iversen, Ptito, Møller, & Kupers, 2015). Moreover, studies of auditory social perception reported that blind participants were able to perform voice-based size estimation as accurately as sighted participants (Pisanski, Oleszkiewicz, & Sorokowska, 2016; Pisanski, Feinberg, Oleszkiewicz, & Sorokowska, 2017). However, it remains unclear which modalities are most important in social assessments performed by blind people, and whether there is a difference compared with the mechanisms of social assessment reported by sighted individuals.

Several previous studies have examined the relative importance of various sensory cues in social assessment, reporting that vision and olfaction were generally highly important in the initial evaluation of strangers (Havlicek et al., 2008; Herz & Cahill, 1997; Herz & Inzlicht, 2002). Interestingly, men and women have been reported to differ in the relative importance attributed to different sensory modalities—while male participants reported that visual cues were the most important in lover choice, female participants reported that olfactory signals were most important (Havlicek et al., 2008; Herz & Cahill, 1997; Herz & Inzlicht, 2002). In addition, Havlicek et al. reported that women considered smell more important also outside of a sexual context. Despite these reported sex differences, it is worth noting that, in all these studies, vision was rated as very important. Thus, the effect of visual impairment on sexual attractiveness assessment (and potential sex differences with this regard) requires further investigation.

The current study was conducted to explore the relative importance of sensory modalities other than vision (i.e., smell, touch, and audition) in assessments of opposite-sex strangers. We specifically focused on possible sensory compensation in the context of mate selection, defined as the enhanced importance of modalities other than vision among blind individuals in their choice of potential partners. Overall, we hypothesized that the importance and reliance on non-visual sensory cues would be higher for blind people compared with sighted individuals. Specifically, we assumed that audition might be more important for blind than sighted people as previous studies show auditory compensation among blind people in social and non-social contexts and low importance of audition in lover choice for sighted people (Havlicek et al., 2008; Herz & Cahill, 1997; Herz & Inzlicht, 2002). As these previous studies showed high importance of olfaction in mate choice, we also assumed that olfaction would be very important for both sighted and blind people.

## Method

### Participants

Data were obtained from a total of 119 heterosexual participants, of whom 78 were blind people aged between 16 and 65 years (*M* = 42.4, SD = 12.6; 38 females) and a control sample of 41 sighted people aged between 20 and 64 (*M* = 39.7, SD = 14.3; 22 females). Among the blind participants, 41 (22 females) were early blind [they were either born blind or had lost their sight before the age of 2, i.e., before completion of visual development (Wiesel, 1982)], and 45 were late blind (20 females). In the late blind group, duration of sight loss ranged from 1 to 58 years (*M* = 26.5). The participants were recruited by a specialized agency that paid the subjects 30 US dollars for participation in a series of short studies. Blind participants were contacted through local associations for blind people in Poland, and sighted participants were found by means of leaflets and press releases. Blind and sighted groups were roughly matched on sex and age.

### Procedure

Data were collected during individual sessions. A trained experimenter instructed each participant that he/she would be asked a series of questions relating to the importance of different modalities in assessments of strangers. To assess overall differences in social sensory assessments between blind and sighted individuals, we asked questions about an opposite-sex person (potential partner) and control questions about assessments of a same-sex stranger. The experimenters explained that a potential partner meant a mate (a romantic or sexual partner). The statements concerning assessments of others were adapted from the Sensory Stimuli and Sexuality Survey (Herz & Cahill, 1997): When meeting a potential partner/same-sex stranger, the way this person sounds (his/her voice) can make a big difference to me; when meeting a potential partner/same-sex stranger, the way this person feels (his/her skin) can make a big difference to me; and when meeting a potential partner/same-sex stranger, the way this person smells can make a big difference to me. The participants rated each modality using a 5-point Likert scale, where 1 meant *definitely not* and 5 meant *definitely yes*. Both sighted and blind participants were read the questions aloud, and the experimenter recorded their responses to the questionnaire.

### Statistical Analyses

We performed an omnibus analysis of variance (ANOVA) with repeated measures design. Within the tested model, we included modality (audition vs. touch vs. smell), and target (potential partner vs. same-sex stranger) as within-subject factors and participants’ sex (male vs. female) and sightedness (blind vs. sighted) as between-subject factors. For multiple comparisons, we undertook Bonferroni corrections. Consequently, based on the observed effects, we performed analogous models for each target separately (see below). Statistical analyses were performed using SPSS v. 21 with *p* < .05 set as the level of significance.

## Results

 Table 1 presents mean importance attached to each modality by sighted and blind women and men. In the course of an omnibus ANOVA, we observed a main effect of target, *F*(1, 115) = 111.7, *p* < .001, *ŋ*^2^ = .49, and its interaction with modality, *F*(2, 230) = 32.7, *p* < .001, *ŋ*^2^ = .22, (all effects for the omnibus model are described in Supplementary File 1). Therefore, we computed two models, for each target separately. We considered effects of sightedness, sex, and modality on assessments of two types of targets–a potential partner and same-sex stranger.

**Table 1:** Mean importance attached to each modality by sighted and blind women and men

	*N*	Statistic	Partner			Same-sex stranger		
			Audition	Touch	Smell	Audition	Touch	Smell
Sighted								
Female	22	*M*	4.27	3.45	4.73	3.50	2.23	4.41
		SEM	0.19	0.23	0.15	0.22	0.25	0.19
Male	19	*M*	3.58	3.95	4.21	3.42	2.16	3.89
		SEM	0.20	0.25	0.16	0.23	0.27	0.21
Overall	41	*M*	3.95	3.68	4.49	3.46	2.20	4.17
		SEM	0.14	0.17	0.11	0.16	0.18	0.14
Blind								
Female	38	*M*	4.53	3.95	4.47	4.39	2.53	4.11
		SEM	0.14	0.18	0.12	0.17	0.19	0.15
Male	40	*M*	4.50	4.10	4.58	3.65	2.58	3.88
		SEM	0.14	0.17	0.11	0.16	0.18	0.14
Overall	78	*M*	4.51	4.03	4.53	4.01	2.55	3.99
		SEM	0.10	0.12	0.08	0.12	0.13	0.10

*SEM* standard error mean

### The Role of the Three Modalities in Mate Selection

**Fig. 1:**
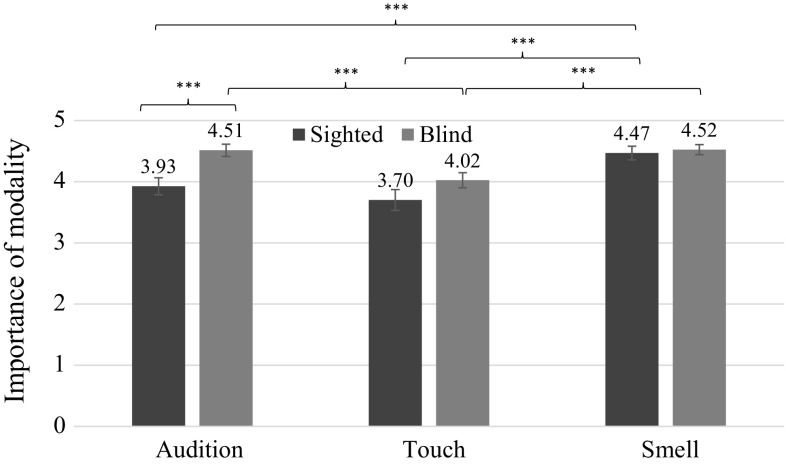
Interaction effect between sightedness and importance attached to the three modalities in the assessments of potential partners (± SE). *Note* ***p ≤ .001

We found a significant interaction between modality and sightedness, *F*(2, 230) = 3.49, *p* = .032, *ŋ*^2^ = .03. Pairwise comparisons indicated that blind people relied more heavily on audition than their sighted counterparts (*p* = .001). Among sighted individuals, smell was significantly more important than the two other modalities (*p*s < .001), while among blind people touch was significantly less important than the two other modalities (*p*s < .001) (see Fig. 1).

Further, we found a significant interaction effect between modality and sex, *F*(2, 230) = 6.34, *p* = .002, *ŋ*^2^ = .05, with pairwise comparisons showing that women valued audition and smell significantly more than touch (*p*s < .001), while for men smell was significantly more important than touch and audition (*p*s < .014). Also, women rated the importance of audition as significantly higher than men (*p* = .038). Critically, we found a significant three-way interaction between modality, sex, and sightedness, *F*(2, 230) = 3.98, *p* = .02, *ŋ*^2^ = .03. The pairwise comparisons showed a significant difference between importance attached to particular modalities—blind men valued audition significantly more than sighted men (*p* < .001) in the mating context.

### The Role of the Three Modalities in Social Assessments

**Fig. 2:**
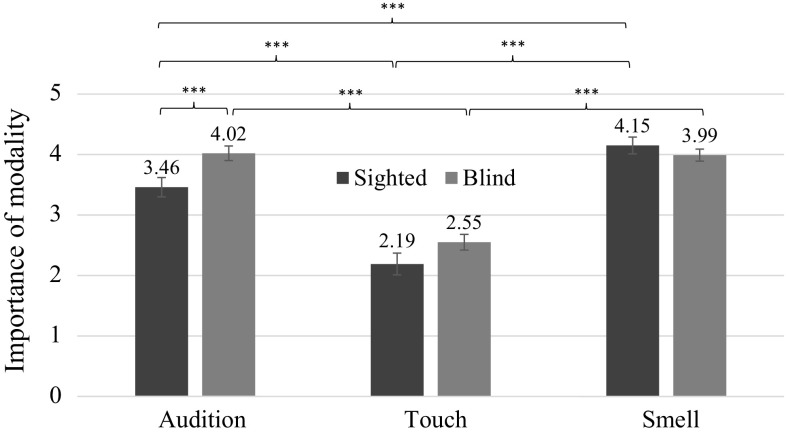
Interaction effect between sightedness and importance attached to the three modalities in the assessments of same-sex stranger (± SE). *Note* ***p ≤ .001

We found a significant interaction between modality and sightedness, *F*(2, 230) = 4.47, *p* = .012, *ŋ*^2^ = .04. Pairwise comparisons indicated that blind people relied more heavily on audition than their sighted counterparts (*p* = .005). Among sighted individuals, smell was significantly more important than audition (*p* = .001), which was more important than touch (*p* < .001), while among blind people touch was significantly less important than two remaining modalities (*p*s < .001; Fig. 2).

## Discussion

In the present study, we examined whether blind men and women perceive modalities other than vision (i.e., smell, touch, and audition) as more important in social assessments and mate choice, compared with sighted men and women. We observed a compensatory effect of blindness on auditory assessments. Our data indicate that visual impairment increases the importance of audition in social assessments (for both sexes) and mating context (for blind men). Detailed analyses of various sensory cues that can be assessed by men and women revealed no significant differences between blind and sighted people in the importance of smell or touch. This means that the relationship between blindness and sensory premises for attractiveness judgments is not just a simple increase in the level of attention paid to all stimuli other than vision.

Interestingly, both previous studies (Havlicek et al., 2008; Herz & Cahill, 1997; Herz & Inzlicht, 2002) and our research demonstrated relatively low importance attributed by sighted men to audition in mate choice. At the same time, audition was valued significantly more by blind men. Given that for men, physical attractiveness is extremely important (Havlicek et al., 2008), in the absence of vision, blind men might rely on auditory cues in mate choice, because voices and visual signals represent reliable and coherent signals of mate quality (for a review, see Puts, Jones, & DeBruine, 2012). Previous studies have reported that faces and voices convey similar information about people’s health (Smith, Dunn, Baguley, & Stacey, 2016), height (Pisanski, Fraccaro, Tigue, O’Connor, & Feinberg, 2014), and personality traits important for mate selection, such as masculinity/femininity (Borkenau & Liebler, 1992; Smith et al., 2016). Also, attractiveness judgments have been reported to covary across these two modalities (Collins & Missing, 2003; Hughes & Miller, 2016; Saxton, Caryl, & Roberts, 2006). Blind men could thus learn to rely on auditory cues to substitute for vision in mate choice, while for sighted men voices are not that important given that easily available visual cues provide similar information. As for the value of auditory cues reported by blind men and women in social assessments, this effect could again be due to voices being a reliable source of information about, for example, personality traits of other people (Borkenau & Liebler, 1992; Smith et al., 2016). Further, faces and voices constitute salient, distal social stimuli (Belin et al., 2004), and at least some distal information is necessary at the early stage of social relationships for people to become acquainted with each other (e.g., when assessments must be made across a physical distance or when people do not know each other very well). In summary, the observed importance of voices for blind people might thus result from a compensatory role audition plays for them in different social relationships with other people.

Nevertheless, the current findings highlight the relative importance of smell in comparison with other sensory cues in mate selection, as olfactory cues were rated as very important by both the blind and the sighted participants. Similarly to audition, this finding may have resulted from the influence of body odor on overall attractiveness judgments (Thornhill et al., 2003). Further, smell provides important information for screening against incompatible mating partners (Wedekind, Seebeck, Bettens, & Paepke, 1995), which again supports the influence of biological cues, and of olfaction in particular, in mate-selection strategies. The present results were in accord with the patterns reported by previous studies (Havlicek et al., 2008; Herz & Cahill, 1997; Herz & Inzlicht, 2002). Both the current data and previous reports indicate the importance of olfactory information for women in both sexual and non-sexual contexts, and for men in choice of a potential lover. Herz and Cahill (1997) suggested that male interest in olfactory information about females during mate choice could result from smell being also relevant to offspring viability. Further, female smell might be indicative of cycle phase/fertility (Cerda-Molina, Hernández-López, Claudio, Chavira-Ramírez, & Mondragón-Ceballos, 2013; Havlíček, Dvořáková, Bartoš, & Flegr, 2006). Our results were consistent with this notion. Also, the high level of importance attributed to the sense of smell in the context of mate assessment is expected, given the effects of the loss of this modality on human relationships. For example, Croy et al. (2013) reported that anosmia can enhance social insecurity; congenitally anosmic men were found to exhibit significantly fewer sexual relationships, while anosmic women were reported to feel less secure about their partners.

It should be noted that the different level of sensory compensation for audition and olfaction in the case of mate selection might have resulted from other factors. Contrary to a number of studies consistently reporting superior auditory processing among blind people (Lessard, Paré, Lepore, & Lassonde, 1998; Lewald, 2002, for a review, see Kupers & Ptito, 2014), results of studies on objective olfactory sensitivity often show that it is not significantly higher among blind people (Guducu, Oniz, Ikiz, & Ozgoren, 2016; Luers et al., 2014; Sorokowska, 2016; for a review, see Kupers & Ptito, 2014), even if they report higher olfactory awareness (Beaulieu-Lefebvre et al., 2011; Ferdenzi et al., 2010) and can discriminate negative emotions based on body odor samples (Iversen et al., 2015). Thus, given similar performance of blind and sighted people in various smell tasks, olfactory sensory compensation may not be particularly pronounced in visual impairment. In this case, the importance of olfactory perception of blind people in mate selection and social assessments might be like that of sighted individuals.

Finally, we observed no significant differences between blind and sighted participants in terms of tactile perception—neither when a potential partner was an assessment target, nor when it was a same-sex stranger. Also, both groups placed the lowest importance on this sensory modality in mate choice. This might have resulted from the fact that physical touch typically conveys much higher intimacy than contact through any other modalities, since the mere act of touching is related to the perception of receptivity/trust, affection, and informality (Burgoon, 1991). In most cases, uninvited touch from a stranger is experienced as intrusive and offensive and might be even perceived as threatening (Sussman and Rosenfeld, 1978). Touching is also much more harassing that verbal contact in the case of colleagues (Gutek, Morasch, & Cohen, 1983). Thus, touch might be too intimate to be involved in assessment at the early stage of a relationship, regardless of a person’s sightedness. Nevertheless, it needs to be noticed that this finding is fully consistent with only one previous study (Havlicek et al., 2008), while other researchers found that touch was more important (Herz & Cahill, 1997) or less important than audition (Herz & Inzlicht, 2002) for men, while for women the importance was very similar (Herz & Cahill, 1997; Herz & Inzlicht, 2002). Therefore, it seems that the role of touch in mate choice deserves further investigation.

A possible limitation to the interpretation of our results resides in the applied procedures. Although self-reports are an extremely popular method in psychological studies, it needs to be noted that in our study we used data collected in a single interview/assessment session. In the absence of any follow-up session, the reliability of subjects’ reports remains unassessed. In addition, although self-reports contain large components of accuracy (Funder, 1995), self-reported responding about mate selection is not necessarily identical to real mate choices (Sorokowski, Sabiniewicz, & Sorokowska, 2015). Future studies in this area should combine self-assessments with real-life behaviors, thus ensuring higher reliability of the observed outcomes. Further, the questionnaires were read aloud by an experimenter and the questions about mate choice may have been perceived as sensitive by some participants. However, we ensured that the conditions for blind and sighted participants were identical; therefore, this should not have affected the magnitude of differences we observed in this study. Finally, it would be also very interesting to examine whether the blindness onset would influence the observed effects, but investigating this problem would require a larger sample size for adequate statistical power in the case of our experimental setup.

### Summary

The current study examined the compensatory role that different modalities might play in mate choice and social assessments in the absence of vision. The data indicated the presence of sensory compensation in audition, but not in smell or touch. There are an estimated 37 million blind people globally, and a further 124 million people with low vision who are at risk of becoming blind (Resnikoff et al., 2004). The current findings provide some insight into how members of this large social group might formulate their assessments of others in relation to mate choice and a broader social context.

## Electronic supplementary material

Below is the link to the electronic supplementary material.

Supplementary material 1 (DOCX 27 kb)
