# Supplementary material for: A Compensatory Effect on Mate Selection? Importance of Auditory, Olfactory, and Tactile Cues in Partner Choice among Blind and Sighted Individuals
Source: Arch Sex Behav. 2018 Feb 2;47(3):597–603. doi: 10.1007/s10508-018-1156-0 (PMC5834579; doi:10.1007/s10508-018-1156-0)
Supplement: Supplementary file 1 — Supplementary material 1 (DOCX 27 kb) [file 10508_2018_1156_MOESM1_ESM.docx]

**Results of the omnibus ANOVA**

An omnibus ANOVA revealed a main effect of target, *F*(1, 115) = 111.7, *p* < .001, *ŋ*^2^ = .49, with pairwise comparisons indicating that each modality was rated as more important for assessments of a potential partner (*M* = 4.2 ± .07) as compared to same-sex stranger (*M* = 3.4 ± .07; *p* < .001). We also found a significant main effect of modality, *F*(2, 230) = 99.9, *p* < .001, *ŋ*^2^ = .47. Pairwise comparisons showed that overall the most important modality was smell (*M* = 4.3 ± .07) that was rated significantly higher than audition (*M* = 4.0 ± .08, *p* <. 001); touch was rated as significantly less important than smell and audition (*M* = 3.1 ± .08, *p* < .001).

Interestingly, we found a significant interaction effect between modality and sightedness, *F*(2, 230) = 6.9, *p* = .001, *ŋ*^2^ = .06. Pairwise comparisons showed that among sighted individuals smell was significantly more important than audition (*p* < .001) and these two were significantly more important than touch (*p*s < .001); among blind individuals smell and audition were rated as equally important and both were rated higher than touch (*p*s < .001). In addition, we observed that audition (*p* < .001) and touch (*p* = .04) were significantly higher for blind than for sighted individuals (see Fig. 1). We also found a significant interaction effect between modality and sex, *F*(2, 230) = 5.7, *p* = .004, *ŋ*^2^ = .05, indicating that, among men, smell was significantly more important than audition (*p* = .01), whereas women reported no significant difference between the importance of smell and audition (*p =* .08), but these two modalities were significantly more important than touch (*p*s < .001). Additionally, smell (*p* = .03) and audition (*p* = .01) were significantly more important for women than for men, whereas no such difference was observed in the case of touch (*p* = .35). We observed a significant interaction effect between target and modality, *F*(2, 230) = 32.7, *p* < .001, *ŋ*^2^ = .22, indicating a stronger effect of modality on judgments of a same-sex stranger than a potential partner. Each modality was significantly more important in assessments of a potential partner than a same sex stranger (*p*s < .001). However, the direction of the relationships was the same for both targets–smell was the most important, followed by audition (*p*s < .02), while touch proved to be the least important (*p*s < .004). Finally we found a significant four-way interaction between sightedness, modality, sex and target, *F*(2,230)=4.6, *p=*.01, *ŋ*^2^ = .04. All ratings can be found in Table 1.

*Figure 1: Interaction effect between sightedness and importance attached to the three modalities in the assessments of both types of targets (±standard error).*

*Note: *** p* <.001; ** *p* < .01; * *p* < .05
